# Prospective audit and feedback on antibiotic use in neonatal intensive care: a retrospective cohort study

**DOI:** 10.1186/s12887-019-1481-z

**Published:** 2019-04-11

**Authors:** Nisha Thampi, Prakesh S. Shah, Sandra Nelson, Amisha Agarwal, Marilyn Steinberg, Yenge Diambomba, Andrew M. Morris

**Affiliations:** 1 Department of Pediatrics, CHEO, 401 Smyth Road, Ottawa, ON K1H 8L1 Canada; 2 Department of Pediatrics, Sinai Health System, 600 University Avenue, Toronto, ON M5G 1X5 Canada; 3 Research Institute, CHEO, 401 Smyth Road, Ottawa, ON K1H 8L1 Canada; 4 Antimicrobial Stewardship Program, Sinai Health System-University Health Network, 600 University Avenue, Toronto, ON M5G 1X5 Canada; 5 Department of Medicine, Sinai Health System, University Health Network, 600 University Avenue, Toronto, ON M5G 1X5 Canada

**Keywords:** Prospective audit and feedback, Antibiotics, Neonatal, Intensive care, Antimicrobial stewardship

## Abstract

**Background:**

Antimicrobial stewardship programs potentially lead to appropriate antibiotic use, yet the optimal approach for neonates is uncertain. Such a program was implemented in a tertiary care neonatal intensive care unit in October 2012. We evaluated the impact of this program on antimicrobial use and its association with clinical outcomes.

**Methods:**

In a retrospective cohort study, we examined 1580 neonates who received antimicrobials in the 13-months before and 13-months during program implementation. Prospective audit and feedback was given 5 days a week on each patient who was receiving antibiotic. Pharmacy and microbiology data were linked to clinical data from the local Canadian Neonatal Network database. The primary outcome was days of antibiotic therapy per 1000 patient-days; secondary outcomes included mortality, necrotizing enterocolitis, and antibiotic duration for culture-positive and culture-negative late-onset sepsis. The breadth of antibiotic exposure was compared using the Antibiotic Spectrum Index.

**Results:**

Overall antibiotic use decreased to 339 days of therapy per 1000 patient-days from 395 (14%, *P* < 0.001), without an increase in mortality. There was no difference in duration of therapy in culture-negative or culture-positive sepsis, rates of necrotizing enterocolitis, or breadth of antibiotic exposure. Fewer antibiotic starts occurred during program implementation (63% versus 59%, *P* < 0.001). The use of narrow-spectrum agents decreased (*P* < 0.001) whereas the use of cefotaxime increased (*P* = 0.016) during program implementation.

**Conclusions:**

Daily prospective audit and feedback was not associated with a change in antibiotic duration or clinical outcomes, however there were fewer babies started on antibiotics, suggesting that additional interventions are required to inform and sustain changes in antibiotic prescribing practices.

## Background

In North American hospitals, antimicrobial stewardship programs (ASPs) have become a required practice for accreditation [1]. While a cohort study of 31 pediatric institutions with formalized ASPs demonstrated significant decreases in antibiotic use, [2] implementation in the neonatal intensive care unit (NICU) setting has had variable success despite strong engagement of neonatal care teams, and different metrics to evaluate prescribing practices [3–6].

Up to 72% of neonates admitted to intensive care are prescribed antibiotics [7, 8] and up to 30% of those antibiotics have been shown to be inappropriate, especially with regard to continuation of therapy [6, 9]. Diagnostic challenges in this population contribute to uncertainty surrounding antibiotic discontinuation, including non-specific signs and symptoms of sepsis, rapid progression to overwhelming sepsis, and concerns of insensitive blood cultures because of small blood volume draws and in utero exposure to maternal antibiotics [10–12]. Against the concern of clinical sepsis are compelling associations between antibiotic overuse and adverse neonatal outcomes, including mortality, late-onset neonatal sepsis, necrotizing enterocolitis (NEC), candidemia, retinopathy of prematurity and bronchopulmonary dysplasia [13–19]. Gut dysbiosis has been demonstrated to persist for at least 4 weeks after treatment; long-term outcomes associated with antibiotic use in early infancy include adverse neurodevelopmental outcomes at 18–21 months corrected age, obesity and asthma [20–23].

In 2009, a multidisciplinary ASP was established at Mount Sinai Hospital in Toronto, Canada; it was expanded through a broader provincial initiative into the NICU in October 2012 [24]. Using prospective audit and feedback (PAF) as the key intervention, we prospectively evaluated antibiotic use among all babies in the NICU pre- and post-implementation of the neonatal ASP, to:

1. Evaluate the impact of the ASP on antimicrobial use in days of therapy per 1000 patient-days, and its association with clinical outcomes, including late-onset neonatal sepsis (LONS), candidemia, NEC and death; and.
2. determine the impact of an ASP on the duration of therapy for culture-positive and culture-negative sepsis.

The study was approved by the hospital’s Research Ethics Board.

## Methods

### Patient population

Our study included neonates admitted to the 55-bed inborn Level II and III NICU at Mount Sinai Hospital, started on an antibiotic and followed until discharge from the NICU. Approximately 1100 newborns are admitted annually to the unit, which cares for non-surgical preterm and sick term neonates. In 2011, prior to introducing the ASP, the rate of mortality was 5.4%; NEC was 1.9%, culture-positive early- and late-onset sepsis were 0.8 and 7.2%, respectively. Neonates with surgical conditions are sent to a tertiary pediatric hospital. Babies who did not receive an antibiotic were excluded from the study.

The pre-intervention period covered September 1, 2011, to October 21, 2012, and October 22, 2012 to November 30, 2013 reflected the ASP implementation period. Empiric therapy for early-onset neonatal sepsis (EONS, less than 72 h of age) was ampicillin and gentamicin; empiric therapy for LONS (more than 72 h of age) was cloxacillin and gentamicin, or vancomycin and gentamicin if a central catheter was present. All neonates received one blood culture when evaluated for sepsis; a second blood culture was drawn to confirm clearance if the initial blood culture was positive. Cefotaxime was added empirically if there was concern of meningitis from the clinical presentation or cerebrospinal fluid analysis.

### ASP program description

Starting in October 2012, a pediatric infectious diseases-trained physician and/or a pharmacist specializing in antimicrobial stewardship performed PAF fives day a week on every neonate who received antibiotics. They participated in dedicated ASP rounds five mornings a week with the NICU medical team, which included staff physicians, fellows, nurse practitioners, residents and students. Neonates who were being considered for antibiotic treatment were not reviewed. The discussions included reviews of the evidence with the team, such as use of serial C-reactive protein for suspected sepsis, or clarithromycin to prevent bronchopulmonary dysplasia; discussions around microbiology and pathophysiology, such as the management of a baby born to a mother with chorioamnionitis with in-utero antibiotic exposure and negative neonatal blood cultures; support of changes in practice by individuals, such as discontinuing antibiotics with negative cultures in the context of clinical sepsis; and affirmed these changes in presentations at staff meetings. The antibiotic courses for babies followed by the infectious diseases service were not reviewed but were included in the analysis. Suggestions were made around antimicrobial use, including, but not limited to, choice of antimicrobial and duration of therapy. Eight months into the program, the frequency of ASP rounds was decreased to 4 days a week.

### Data

Antibiotic use was determined using days of therapy (DOT) and antibiotic days. DOT was calculated as the number of days the neonate was on a specific agent. For example, ampicillin for 3 days resulted in 3 DOT, irrespective of number of doses received in one day. An antibiotic day is defined as exposure to antimicrobials on a specific day (based on calendar day) and does not take into consideration the number of antimicrobials. Thus, a neonate who received ampicillin and gentamicin for 3 days would have 3 antibiotic days but 6 DOT.

Episodes were based on a count of consecutive dispense dates at the patient level and defined as suspected sepsis if antibiotic days were less than or equal to 3 to reflect empiric therapy pending culture results. If antibiotics were continued for 4 or more days, this was counted as an episode of culture-positive or culture-negative sepsis, depending on the laboratory result that accompanied the initial dispense date. If there were 3 or more days between dispense dates, then it was assumed to be a new episode.

Antimicrobial data were obtained from the Mount Sinai Hospital pharmacy database (PharmNet®, Cerner Corporation, North Kansas City, MO) for each patient, and included the name of the agent, the route of administration and the date(s) dispensed. Topical antibiotics, prophylactic antibiotics, immunizations, antifungals, antivirals and anti-retrovirals were excluded from the analyses. Patient-level pharmacy data were linked to microbiological data from the laboratory information system (SoftMic®, SCC Soft Computer, Clearwater, FL). Data on all positive cultures from sterile sites during the neonate’s admission were collected. These were also linked to clinical data extracted from the Canadian Neonatal Network (CNN) database, which uses standardized definitions to capture information from neonate medical records [25]. Study variables gathered from the database included gestational age, birthweight, mortality, diagnosis of NEC as modified Bell stage 2 or greater, and the Score for Neonatal Acute Physiology, version II (SNAP-II), a validated measure of severity of illness in a newborn during the first 24 h of admission to the NICU [18, 26, 27].

To assure data quality and adequacy, the data abstractor adhered to definitions from the CNN’s Abstractor’s Manual for clinical characteristics and outcomes of interest [25]. The database has been shown to have high reliability and accuracy [28]. Pharmacy data were repeatedly measured during and after the study period, and microbiology data were extracted by 2 independent sources to check for completeness and accuracy. Once the data were linked from the pharmacy, microbiology and CNN databases, they were manually reviewed by 2 study authors to assure data quality.

### Outcomes

Our primary outcome measure was antibiotic DOT per 1000 patient-days (the sum of the DOT accounting for each antibiotic over 1000 patient-days). Secondary outcomes were proportion of neonates who received antibiotics during their admission; number of antibiotic days and total length of stay; mortality prior to discharge; NEC stage 2 or 3; duration of therapy for LONS, defined as an antibiotic course of 3 or more days in a symptomatic neonate aged 3 days or greater; and the incidence of antibiotic restarts within 3 days after discontinuation, as a balancing measure. We also examined duration of therapy — i.e. total consecutive antibiotic days — for culture-positive and culture-negative sepsis episodes.

We evaluated the breadth of antibiotic exposure using the Antibiotic Spectrum Index (ASI), which classifies antibiotics according to spectrum of activity, and has been shown to be sensitive to antibiotic selection for specific conditions, as compared to DOT per 1000 patient-days [29]. Using this metric, each antibiotic was scored from 1 to 13 (1 being the most narrow-spectrum agent and 13 being the most broad-spectrum agent), and a composite score of ASI per antibiotic day per patient was obtained for culture-confirmed bacteremia and culture-negative LONS.

### Analysis

Clinical characteristics were summarized using descriptive statistics; the Mann-Whitney U test and Pearson’s chi-squared test or Fisher’s exact test was used, as appropriate, for significance testing between the two periods.

Antibiotic DOT per 1000 patient-days pre- versus during ASP was analyzed using a Poisson model, and 95% confidence intervals were reported. This was done for the overall comparison and subgroups. Two-sided *p*-values less than 0.05 were considered statistically significant. All statistical analyses were performed using R statistical software version 3.4.2 (R Core Team, Vienna, Austria) [30].

## Results

Birthweight, gestational age and SNAP II scores on admission were similar between the pre- and during ASP groups. The length of stay and number of antibiotic days between the groups were similar pre-ASP and during the implementation period (Table 1). Rates of mortality, Stage 2 or 3 NEC and culture-positive sepsis did not differ between the two groups. There was no difference in the overall incidence of culture-negative LONS, although a higher proportion of neonates aged 29–33 weeks gestational age received antibiotics for at least 4 days, despite negative culture results, during the ASP period. There was no difference in duration of therapy for culture-negative sepsis; neonates treated for EONS received a mean of 6 days of antibiotics in both periods, and neonates treated for LONS received a mean of 6 days (interquartile range 4–7 days) compared to 7 days (IQR 4–8 days) in the pre- and during ASP periods, respectively.

**Table 1:** Characteristics and clinical outcomes of neonates who received antibiotics pre- and post-ASP implementation

Characteristic	Pre-ASP cohort (*N* = 864)	During ASP cohort (*N* = 716)	*p*-value
Birth weight (grams), median (IQR)	1960 (1223, 2888)	1920 (1180, 2910)	0.49
Gestational age (weeks), median (IQR)	33 (29, 37)	33 (29, 37)	0.55
SNAP II score, median (IQR)	10 (5, 19)	10 (5, 17)	0.19
Length of stay, median (IQR)	9 (4, 23)	8.5 (4.0, 25.0)	1.00
Antibiotic-free days, median (IQR)	6.0 (1.0–21.0)	6.0 (1.0–24.0)	1.00
Death, N (%)	32 (3.7)	27 (3.8)	1.00
Necrotizing enterocolitis, N (%)	13 (1.5)	19 (2.7)	0.16
Candidemia, N (%)	1 (0.1)	1 (0.1)	1.00
Culture-positive bacterial sepsis	83 (9.6)	64 (8.9)	0.64
Number of episodes^a^, N (%)	103 (9.3)	77 (8.3)	0.45
Episodes of bacteremia, N (%)	58 (56.3)	35 (45.4)	0.15
CoNS (N isolates)	28	21	0.33
Antibiotic days, median (IQR)	10.0 (7.0, 13.2)	9.0 (7.0,12.0)	0.24
*Escherichia coli* (N isolates)	11	7	0.41
Antibiotic days, median (IQR)	17.0 (3.0,20.0)	16.0 (5.5,20.0)	0.89
Culture-negative late-onset sepsis, N (%)	58 (6.7)	56 (7.8)	0.40
Number of episodes ^a,b^, N (%)	192 (17.4)	179 (19.4)	0.30
GA ≤ 28 weeks, N (%)	124 (64.6)	113 (63.1)	0.86
GA 29–33 weeks, N (%)	33 (17.2)	43 (24.0)	0.15
GA ≥ 34 weeks, N (%)	35 (18.2)	23 (12.8)	0.19

^a^There were 1096 episodes pre-ASP and 907 episodes during the ASP period

^b^There was no GA information for 6 neonates in the pre-ASP period and 3 neonates in the post-ASP period

*ASP* antimicrobial stewardship program, *CoNS* coagulase-negative *Staphylococcus* species, *GA* gestational age, *IQR* interquartile range

There were 2582 neonates admitted to the NICU during the study period; 1580 (61%) were started on antibiotics at some time during their stay (864 pre-ASP and 716 post-ASP). Following ASP implementation, antibiotic consumption decreased by 56 DOT per 1000 patient-days (14%) among all gestational age groups, most significantly among extreme premature and near-term neonates (Table 2). There was also a significant reduction in proportion of neonates who received antibiotics (overall 63% versus 59%, *P* < 0.001), particularly in the 29–33 weeks gestational age group.

**Table 2:** Cohort of neonates who received antibiotics and consumption patterns during hospitalization

	Pre-ASP Sept 1, 2011-Oct 21, 2012			During ASP Oct 22, 2012-Nov 30, 2013		
Gestational age (weeks)	Admissions	Received antibiotic(s)	DOT per 1000 patient-days	Admissions	Received antibiotic(s)	DOT per 1000 patient-days
Overall	1367	864 (63.2)	395	1215	716 (58.9) *	339**
≤ 28	198	183 (92.4)	391	177	163 (92.1)	322**
29–33	312	310 (99.4)	376	298	168 (56.4)**	354
≥ 34	857	421 (49.1)	414	740	326 (44.1)	364**

*P < 0.05

**P < 0.001

*ASP* antimicrobial stewardship program, *DOT* Days of therapy

**Fig. 1:**
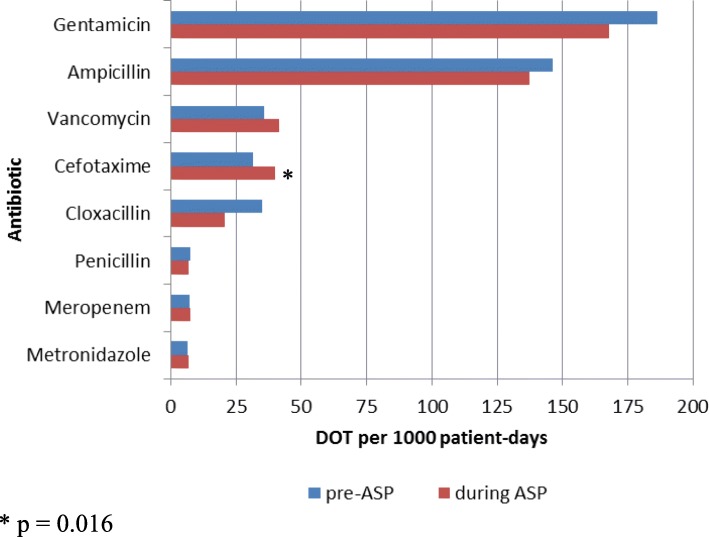
Antibiotic use before and during ASP implementation, by antibiotic, reported as DOT per 1000 patient-days. ASP: antimicrobial stewardship program. DOT: days of therapy

Gentamicin, ampicillin, cefotaxime and vancomycin were the most frequently prescribed antibiotics, reflecting 87 and 90% of antibiotics in the pre- and during ASP implementation periods, respectively (Fig. 1). There were decreases in the use of ampicillin, gentamicin, and cloxacillin (*p* < 0.001), and an increase in the use of cefotaxime (*p* = 0.016) and non-significant increase in the use of vancomycin (*p* = 0.353) during ASP implementation.

Among the neonates with culture-positive bacteremia, three neonates in the during ASP group had the same organism isolated (two with *Streptococcus agalactiae*, one with *Escherichia coli*) within 4 to 6 days of discontinuing antibiotics. Bacteremia occurred more frequently in the pre-ASP period, although not statistically significant (Table 1). There was no difference in antibiotic days for coagulase-negative *Staphylococcus* species or *E.coli* bacteremia, the two most common pathogens in the unit. There was no change in the spectrum of antibiotic use following ASP implementation; ASI scores did not differ significantly in the treatment of culture-positive bacteremia due to Gram-negative and Gram-positive organisms, nor for culture-negative LONS (Table 3).

**Table 3:** Antibiotic spectrum index patterns across indications^a^

Characteristic	Pre-ASP		During ASP		
	N	Median (IQR)	N	Median (IQR)	*p*-value
Culture-positive bacteremia	60	7.2 (5.8, 8.5)	32	6.9 (6.3, 7.9)	0.79
Gram-negative organism	23	8.0 (7.5, 10.8)	9	7.8 (6.8, 10.0)	0.66
Gram-positive organism	46	6.5 (5.4, 8.0)	23	6.6 (5.6, 7.5)	0.90
Culture-negative late-onset neonatal sepsis	58	7.7 (6.0, 10.0)	60	8.3 (6.1, 10.0)	0.43

^a^Patients who died or were transferred within 3 days of starting antibiotics for culture-positive infection were excluded

*ASP* antimicrobial stewardship program, *IQR* interquartile range

## Discussion

Daily PAF in a Level III NICU was associated with an overall decrease in antibiotic consumption of 14%. There were no differences in neonatal characteristics and no changes in the microbiology lab testing methods during the study period to otherwise explain this reduction in antibiotic use. Consumption was significantly reduced among extremely preterm and near-term neonates, the latter of whom were also less frequently started on antibiotics. There was no change in the proportion of neonates continued on antibiotics despite negative culture results, and no significant change in duration of therapy for culture-positive infections. While there was a significant decrease in the use of narrow-spectrum antibiotics during the ASP implementation period, there was a significant increase in cefotaxime use.

Studies that examined the impact of PAF in the NICU setting had varying conclusions of its effectiveness in improving antibiotic prescribing practices [3–6, 31]. A significant decrease in antibiotic use was seen in settings where PAF was preceded by a review of antibiotic prescribing practices, which then informed guideline development and education to neonatology staff and trainees prior to PAF implementation [3, 4]. On the other hand, a recent quasi-experimental study that included PAF showed no significant difference in total antibiotic DOT per 1000 patient-days, although there were significantly fewer evaluations and antibiotic initiation events for LONS [5]. Similarly, the addition of PAF to ongoing education and guideline implementation did not decrease the use of vancomycin in another NICU setting [31]. Our findings suggest that PAF alone is limited in influencing antibiotic prescribing behaviours, particularly around discontinuation of antibiotics in neonates, although there were fewer antibiotic starts. While the ASP was designed to provide feedback after antibiotics were initiated, the decrease in antibiotic starts suggests that the daily discussions around antibiotic indications may have affected the decision to initiate antibiotics.

The decrease in narrow-spectrum antibiotic use and increase in broad-spectrum antibiotics seen in this study may reflect a phenomenon previously described as “squeezing the balloon,” an unintended consequence of managing one component of antibiotic overuse [32]. Although initially described where restriction of cephalosporins was followed by an increase in imipenem use and imipenem-resistant *Pseudomonas aeruginosa*, this phenomenon has also followed other ASP implementation efforts, and offers an explanation for the lack of change in antibiotic days despite a decrease in DOTs, as ampicillin and gentamicin may have been switched for cefotaxime [3, 33, 34].

There is no clear consensus on the appropriate outcome metrics to study the impact of an ASP in the NICU [35]. The most commonly used metric of DOT per 1000 patient-days can be problematic, particularly among extremely premature neonates, for whom the DOTs are measured against an extended length of stay that results in lower antibiotic utilization rates; conversely, mortality can inflate this metric. Another unique challenge is in the diagnosis and management of EONS. Neonates born to mothers with suspected sepsis or intra-amniotic infection have a six-fold increased risk of developing sepsis in the first 72 h after birth [36]. Given the failure of maternal therapy in preventing neonatal sepsis, clinical practice has favoured prolonged antibacterial treatment of neonates born to mothers with confirmed or suspected infection who present with abnormal laboratory findings, despite sterile blood cultures [37]. As such, there is a lack of evidence and rapid diagnostic tools to support antibiotic discontinuation in this common clinical setting.

We examined the Antibiotic Spectrum Index (ASI) in the context of culture-positive bacteremia and culture-negative LONS, and identified no difference in the burden of antibiotic exposure following ASP implementation for both clinical conditions. This metric illustrated a significant decrease in the spectrum of antibiotic prescribing patterns for pediatric community-associated pneumonia as compared to DOT per 1000 patient-days, following an ASP intervention that led to a change in prescribing practice from ceftriaxone (ASI = 5) to ampicillin (ASI = 2) [29]. The ASI metric may be less beneficial in the neonatal context, however. In our NICU, 4 antibiotics constituted up to 90% of antibiotics prescribed during the study period. With an ASI of 6 for first-line therapy for LONS (cloxacillin ASI = 1 and gentamicin ASI = 5), compared with vancomycin or cefotaxime (ASI = 5), this metric does not offer enough discrimination to measure the impact of an ASP on LONS. Similarly, in EONS, lower ASI values are found with broad-spectrum therapy. Moreover, the ASI may not be feasible, as it requires manual calculation when multiple antibiotics are prescribed over variable antibiotic days within one episode of suspected or confirmed infection.

### Strengths and limitations

The strengths of this study lie in its multi-disciplinary involvement with an infectious diseases physician and ASP pharmacist, and incorporation of PAF into the morning clinical rounds, which provided an opportunity for active engagement of neonatal staff and trainees in the discussions. Objective clinical measures were used, as well as a standardized method of reporting antibiotic consumption. Despite being a single institution study, the setting reflects many other Level III NICUs, with a broad mix of inborn non-surgical patients of variable gestational ages.

A number of limitations relate to measurement tools and the study intervention. Dispense data were acquired at the patient level, which may not have been correlated with patient administration; however, we have no reason to believe that this changed between the study periods. We did not document the content of our advice, whether recommendations were agreed upon, and associated clinical outcomes. Review of this information may have helped the ASP to target specific prescribing behaviors using other modalities in addition to PAF. There was also variability in the nature of the PAF discussions based on the readiness of each staff neonatologist to change his/her prescribing practices, which may have affected the duration of therapy and antibiotic choice at various time points.

The clinical context surrounding antibiotic courses was not available for analysis. For example, cerebrospinal fluid studies were not included, so a patient may have appropriately received cefotaxime in the context of suspected meningitis and analyzed as “culture-negative sepsis”, alongside other cases of inappropriately prolonged cefotaxime therapy. Such information would help to determine if the increase in cephalosporin use during the ASP implementation period was appropriate for the clinical context.

## Conclusions

The NICU ASP, with daily prospective audit and feedback by a multidisciplinary team, was associated with a significant decrease in antibiotic consumption despite a lack of significant change in antibiotic duration of culture-negative sepsis and an increase in cefotaxime use. Our findings suggest that PAF alone cannot inform antibiotic prescribing behaviours around discontinuation of antibiotics in neonates. There is a need to define and measure appropriateness of antibiotic use that incorporates clinical context, diagnostic tools, antibiotic choice and duration of therapy into real-time audit and feedback. Such a metric should be generalizable, able to discriminate the risk of bacterial sepsis from other non-infectious complications, particularly in preterm infants, and inform strategies to reduce antibiotic misuse in critically ill neonates.

## Abbreviations

ASI: Antibiotic Spectrum Index
ASP: Antimicrobial stewardship program
CNN: Canadian Neonatal Network
DOT: Days of therapy
EONS: Early-onset neonatal sepsis
LONS: Late-onset neonatal sepsis
NEC: Necrotizing enterocolitis
NICU: Neonatal intensive care unit
PAF: Prospective audit and feedback
SNAP-II: Score for Neonatal Acute Physiology, version I
